# Clinical and Prognostic Significance of a Squamous Cell Carcinoma Component in Endometrioid Endometrial Carcinoma: A Multicenter Retrospective Cohort Study

**DOI:** 10.3390/cancers18142275

**Published:** 2026-07-15

**Authors:** Xiaohang Yang, Huixing Yan, Xinyue Ma, Chang Liu, Zhongshao Chen, Zhaoyang Zhang, Haocheng Zhang, Beihua Kong, Jingying Chen

**Affiliations:** 1 Department of Obstetrics and Gynecology, Qilu Hospital, Cheeloo College of Medicine, Shandong University, Jinan 250012, China; 2 Shandong Key Laboratory of Reproductive Health and Birth Defects Prevention and Control, Shandong University, Jinan 250012, China; 3 Gynecologic Oncology Key Laboratory of Shandong Province, Qilu Hospital, Shandong University, Jinan 250012, China

**Keywords:** endometrial endometrioid carcinoma, squamous cell carcinoma, risk stratification, morphology, prognosis, propensity score, machine learning, SHAP

## Abstract

Background/Objectives: Contemporary endometrial cancer pathology integrates histological and molecular features for risk classification. Endometrioid endometrial carcinoma (EEC) with a squamous cell carcinoma (SCC) component was historically termed adenosquamous carcinoma but later incorporated into the squamous-differentiation subtype of EEC. This reclassification left an evidence gap, and its prognostic significance remains insufficiently defined. We compared clinicopathological aggressiveness and evaluated associations of an SCC component with overall survival (OS) and lymph node (LN) involvement. Methods: We retrospectively analyzed 7590 surgically staged patients from 24 institutions (2000–2019): 7495 pure EEC and 95 EEC with SCC components. Confounding was addressed using overlap weighting (OW), inverse probability of treatment weighting targeting the average treatment effect on the treated (IPTW-ATT), and 1:4 propensity score matching (PSM), each with doubly robust Cox regression. Survival machine-learning models with SHapley Additive exPlanations (SHAP) assessed histological subtype contribution. LN involvement was assessed by OW/IPTW-ATT-weighted logistic regression. Results: EEC with SCC component had higher grade, deeper myometrial invasion, more advanced stage, and worse unadjusted OS (hazard ratio [HR] 7.04; 95% confidence interval [CI] 3.50–14.16; *p* < 0.001). After adjustment, the OS disadvantage persisted: OW-adjusted HR 3.05 (95% CI, 1.36–6.85; *p* = 0.007), IPTW-ATT-adjusted HR 3.26 (95% CI, 1.51–7.01; *p* = 0.003), and PSM-adjusted HR 3.30 (95% CI, 1.24–8.77; *p* = 0.017). XGBoost-Survival showed stable discrimination (test C-index 0.797 ± 0.057; 5-year AUC 0.787 ± 0.065), and SHAP supported histological subtype as a prominent risk-associated contributor after class-imbalance correction. In contrast, adjusted LN involvement was not increased (OW odds ratio [OR] 1.000, 95% CI 0.505–1.979; *p* = 1.000; IPTW-ATT OR 1.035, 95% CI 0.527–2.035; *p* = 0.920). Conclusions: EEC with SCC component was associated with worse OS after adjustment, without an adjusted increase in LN involvement. These findings support explicit morphologic reporting and future validation incorporating systematically recorded molecular testing when available.

## Simple Summary

Endometrioid endometrial carcinoma is usually evaluated using microscopic appearance and, increasingly, molecular information. However, molecular testing is not always completed in routine practice. We studied whether a squamous cell carcinoma component within endometrioid endometrial carcinoma identifies a higher-risk subgroup. In a cohort of 7590 surgically treated patients from 24 Chinese institutions, this component was present in 95 patients and was associated with aggressive tumor features and poorer overall survival after adjustment for available clinical and pathological factors. It was not associated with an adjusted increase in lymph node involvement, suggesting that it should not by itself justify more extensive nodal surgery. These findings support clearer reporting of this component in pathology reports and future studies incorporating molecular data when available.

## 1. Introduction

Endometrial carcinoma is the most common female genital tract malignancy in high-income countries and one of the six most common cancers in women worldwide [1]. In China, it is the second most common gynecologic malignancy, affects younger women, and is rising [2]. Standard management combines surgical staging with risk-adapted adjuvant therapy, and risk stratification reflects age, histology, grade, stage, and lymph-vascular space invasion (LVSI) [3]. Contemporary ESGO/ESTRO/ESP risk stratification and the 2023 FIGO staging framework increasingly integrate clinicopathological factors with molecular classification, but routinely reported morphologic features remain important when complete molecular testing is unavailable [4,5].

The World Health Organization (WHO) classification remains the pathological reference standard and has changed substantially over time. In WHO 2003, endometrial carcinomas were classified mainly by morphology and often simplified into endometrioid and non-endometrioid tumors [6]. Endometrioid carcinoma of the uterine corpus (endometrioid endometrial carcinoma [EEC]), the prototypical estrogen-associated subtype, shows variable glandular, papillary, and solid architecture [7]. WHO 2003 also listed a “variant with squamous differentiation” within endometrioid carcinoma [6]. Squamous differentiation is common, occurring in about 25% of cases, and is recognized by morules, intercellular bridges, keratin pearls, and solid eosinophilic polygonal cells [8,9]. Benign-appearing and malignant squamous elements were historically labeled adenoacanthoma and adenosquamous carcinoma, but these terms were criticized for poor reproducibility [9]. Abeler and Kjørstad therefore proposed the more descriptive diagnosis “adenocarcinoma with squamous cell differentiation” [10], yet prognostic reports remained conflicting [9,11,12,13]. WHO 2014 retained “endometrioid carcinoma with squamous differentiation” (International Classification of Diseases for Oncology [ICD-O] 8570/3) within the endometrioid category [14], where squamous differentiation generally parallels glandular differentiation and does not determine grade or stage. ProMisE and TCGA showed stronger prognostic discrimination by polymerase epsilon (POLE)-ultramutated, mismatch repair (MMR)-deficient, TP53-mutant, and no specific molecular profile (NSMP) groups [15,16]. WHO 2020 therefore emphasized integrated morphologic–molecular classification while retaining squamous differentiation, including morules, as an optional descriptor within EEC [17].

A key classification and risk-assessment inconsistency remains. WHO 2020 also recognizes primary squamous cell carcinoma (SCC) of the endometrium (ICD-O 8070/3) among “other endometrial carcinomas” and notes stage-dependent behavior for squamous and mucinous carcinomas [17]. Case reviews report variable but often unfavorable outcomes [18,19,20]. Thus, an SCC component within EEC should not automatically be considered prognostically neutral. Benign squamous differentiation may parallel glandular grade but extending that assumption to a report-defined carcinoma-level SCC component is biologically counterintuitive. Molecular testing remains incomplete in many settings; microsatellite instability (MSI) testing was reported in only 41.5% of Chinese patients [2], keeping morphology-based risk recognition clinically relevant.

Prior EEC-with-SCC studies, historically labeled adenosquamous carcinoma, were mostly small, single-center series [10,12,13,21,22], with inconsistent results and limited adjustment for high grade, deep myometrial invasion, and advanced stage [12]. Thus, its adjusted outcome association and potential contribution to risk assessment remain uncertain. Propensity score (PS) and machine-learning survival methods can reduce confounding and clarify predictor importance [23].

We therefore evaluated whether an SCC component within EEC is associated with OS and LN involvement in a large multicenter cohort, and whether this histological feature contributes prognostic information beyond routinely available clinicopathological variables.

## 2. Materials and Methods

### 2.1. Study Design and Population

This retrospective multicenter cohort included surgically treated patients with primary endometrial carcinoma from 24 Chinese institutions between January 2000 and December 2019. Surgical staging included hysterectomy, bilateral salpingo-oophorectomy, selective lymph node assessment, and peritoneal washing. Adjuvant therapy was selected by center gynecologic oncologists according to routine risk-adapted management. Demographic, clinicopathological, treatment, and outcome data were entered into an anonymized database. The International Federation of Gynecology and Obstetrics (FIGO) 2009 system was applied, with pre-2009 restaging [24]. Pathologic classification followed WHO 2014, with retrospective reclassification of pre-2014 cases. Approval was obtained from the Institutional Review Board of Qilu Hospital of Shandong University (KYLL-202406-042); the study followed the Declaration of Helsinki and received a consent waiver. Data collection at each participating center was conducted using a unified standardized case-report form. At each participating center, two gynecologic-oncology physicians performed source-document abstraction, data entry, and cross-checking. For histopathological variables, the source document was the official institutional final pathology report; entries were verified for consistency across local pathology query systems, inpatient medical-record systems, and hospital case-record systems. After local submission, the records were integrated into a unified dataset. At least two physicians who had a gynecologic-oncology background and had received training in oncologic pathology independently reviewed the electronic registry fields, queried inconsistencies when needed, and reconciled discrepancies. Therefore, the central pathology-related process was centralized electronic field-level quality control and standardized pathology-report-based reclassification. The unit of review was the registry field and its source final pathology report, rather than the glass slide or digital slide; accordingly, we describe this as field-level quality control rather than a slide-level pathology adjudication study.

**Figure 1:**
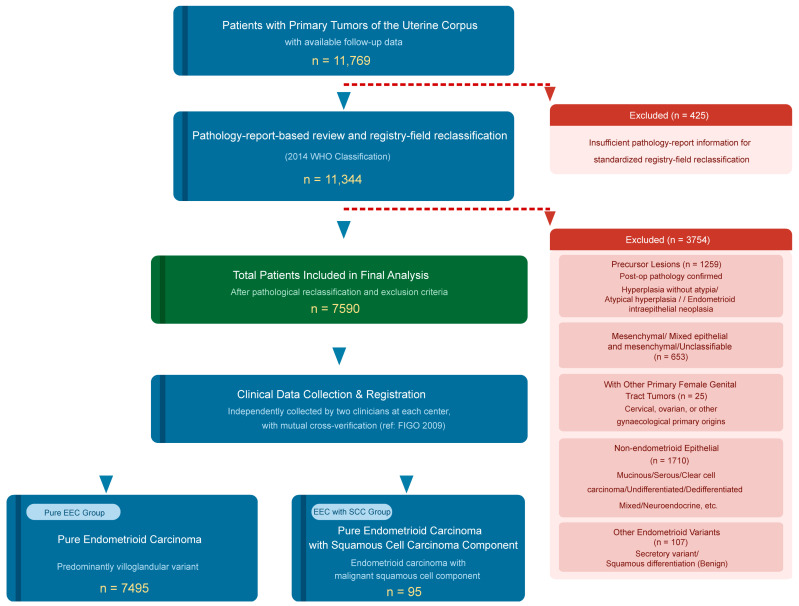
Patient screening and inclusion flowchart.

Eligible patients had histologically confirmed primary endometrial carcinoma, complete follow-up and survival data, and final classification as either pure EEC or EEC with SCC component according to WHO 2014. Pure EEC included conventional EEC with usual or villoglandular architecture but excluded cases with benign squamous or secretory differentiation. EEC with SCC component was defined as otherwise endometrioid carcinoma with an explicitly recorded squamous cell carcinoma component in the institutional final pathology report and retained after standardized registry-field mapping. Historical adenosquamous carcinoma and reports stating endometrioid carcinoma/EEC with a squamous cell carcinoma component were mapped to EEC with SCC component. Reports described only as adenoacanthoma, benign squamous differentiation, squamous metaplasia, morular differentiation, morules, or metaplasia-only wording were not classified as EEC with SCC component. SCC-component proportion, grading, or scoring was not a registry data element, so the exposure was treated as a binary report-based variable. IHC staining intensity or positive-cell percentage for squamous markers was not used as a uniform study-level confirmation criterion because abstraction relied on the final diagnostic report rather than centralized reassessment of primary stains. The standardized mapping of historical pathology-report terms to analytic histological categories is provided in Supplementary Table S1. After exclusions, 7590 patients were included (7495 pure EEC; 95 EEC with SCC component) (Figure 1).

### 2.2. Follow-Up and Outcomes

Follow-up occurred every 3–6 months for 2 years, every 6 months during years 3–5, then annually; imaging was clinically indicated. OS was surgery-to-death from any cause; survivors were censored at last contact. Median follow-up was 44 months (IQR 26–70). Death occurred in 81 patients (1.1%): 9/95 (9.5%) with SCC component and 72/7495 (1.0%) with pure EEC. Median follow-up was 43 months (IQR 26–70) in pure EEC and 70 months (IQR 30–113) in EEC with SCC component.

### 2.3. Baseline Characteristics

Continuous variables are mean ± SD and were compared by Mann–Whitney U test; categorical variables are *n* (%) and were compared by Pearson’s chi-squared or Fisher’s exact test. Covariate imbalance was quantified with absolute standardized mean differences (SMDs): Cohen’s d for continuous variables, pooled-proportion SMD for binary variables, and Mahalanobis distance-based SMD for multicategory variables. Absolute SMD ≥0.1 indicated imbalance. Unknown values indicate unavailable or indeterminate entries in the retrospective multicenter registry and were retained as an Unknown category for categorical covariates rather than handled by complete-case deletion when used in univariate Cox analyses, propensity-score/doubly robust analyses, and exploratory ML/SHAP analyses. This pragmatic missing-category approach preserves transparency in large registry-based analyses but is not a substitute for complete data [25,26].

### 2.4. Univariate Cox Regression

Univariate Cox regression was performed for 29 baseline variables to identify OS associations and guide covariate selection. Missing categorical covariate values were retained as “Unknown.” Results are unadjusted HRs with 95% CIs and two-sided *p*-values.

### 2.5. Propensity Score Estimation

A PS for EEC with SCC component versus pure EEC used 18 prespecified covariates, including diagnosis year for temporal imbalance and clinically relevant risk-stratification variables. Five post-treatment and five symptom variables were excluded to avoid overadjustment/recall bias; ovarian preservation was excluded because it was not prognostic. Variance inflation factors (VIFs) were <3.0 (maximum 2.64) (Supplementary Table S2). Covariate selection is in Supplementary Figure S1.

### 2.6. Propensity Score Adjustment and Doubly Robust Estimation

Three PS methods were used: OW for the average treatment effect in the overlap population (ATO), IPTW-ATT, and 1:4 nearest-neighbor PSM. Balance was checked with Love plots; weighted/matched Kaplan–Meier curves were generated. Implementation details, including trimming/winsorization, caliper selection, and matching specifications, are provided in the Supplementary Methods.

For each method, doubly robust (DR) Cox models [27] combined PS weighting or matching with pathological stage, tumor grade, myometrial invasion depth, and age. Results are aHRs with 95% CIs and two-sided *p* values. Robust variance implementation details are provided in the Supplementary Methods.

To test whether EEC with SCC component was associated with LN metastasis after adjustment, OW and IPTW-ATT weights were applied to PS-weighted logistic regression. Patients with unknown LN status were excluded from this specific binary endpoint analysis (36 patients). Results are ORs with 95% CIs and two-sided *p* values.

### 2.7. Machine Learning Survival Models and SHAP

The machine-learning and SHAP analyses were exploratory interpretability analyses intended to assess whether histological subtype contributed to model-based risk ranking under severe class imbalance; they were not developed or validated as deployable clinical prediction tools. After excluding five symptom variables, 25 clinical variables were used. Missing values were encoded as numeric “Unknown.” Class imbalance (EEC with SCC component:pure EEC = 1:78.9) was addressed by inverse-frequency weighting (78.89 vs. 1.00). Stratified 5-fold cross-validation used subtype and OS event.

CoxPH, Lasso-Cox, RSF, GBSA, and XGBoost-Survival were trained. Performance used Harrell’s C-index, time-dependent area under the ROC curve (AUC), and Brier score. XGBoost-Survival SHAP values used exact TreeSHAP [28,29]. Class-imbalance dilution was examined by gradient downsampling across six pure EEC ratios (original 79:1 and downsampled ratios from 60:1 to 10:1); the original full-cohort ratio was analyzed once, and each downsampled ratio was repeated 10 times. Fold stratification, class-imbalance handling, downsampling implementation, and performance-metric definitions are provided in the Supplementary Methods.

### 2.8. Software

Analyses used Python v3.13 (pandas v2.3, SciPy v1.17, lifelines v0.30, statsmodels v0.14, scikit-learn v1.8, scikit-survival v0.27, XGBoost v3.1, shap v0.50) and R v4.5 (MASS v7.3, WeightIt v1.5, MatchIt v4.7, cobalt v4.6, survminer v0.5, survival v3.8, sandwich v3.1, lmtest v0.9). All tests were two-sided; *p* < 0.05 indicated significance.

## 3. Results

### 3.1. Patient Characteristics

After exclusions, 7590 patients remained: 7495 (98.7%) with pure EEC and 95 (1.3%) with SCC component (Table 1). The SCC-component group was older (*p* = 0.024), more often postmenopausal (*p* = 0.028), and more often had G3 differentiation (37.9% vs. 14.6%; *p* < 0.001), deep myometrial invasion (48.4% vs. 25.0%; *p* < 0.001), FIGO stage III/IV disease (22.1% vs. 11.2%; *p* = 0.001), laparotomy (81.1% vs. 45.0%), and adjuvant therapy (60.0% vs. 38.9%), confirming confounding.

### 3.2. Crude Overall Survival Associations

Univariate Cox results are shown in Table 2. Worse OS was associated with FIGO stage III/IV (HR 7.14), LN positivity (HR 5.54), deep myometrial invasion (HR 4.63), G3 differentiation (HR 4.14), and older age (HR 1.05/year; all *p* < 0.001). Laparotomy was also adverse (HR 4.33; *p* < 0.001), whereas abnormal vaginal bleeding was protective (HR 0.47; *p* = 0.003). EEC with SCC component carried the highest crude mortality risk (HR 7.04; 95% CI 3.50–14.16; *p* < 0.001).

### 3.3. Adjusted Overall Survival Analyses

**Figure 2:**
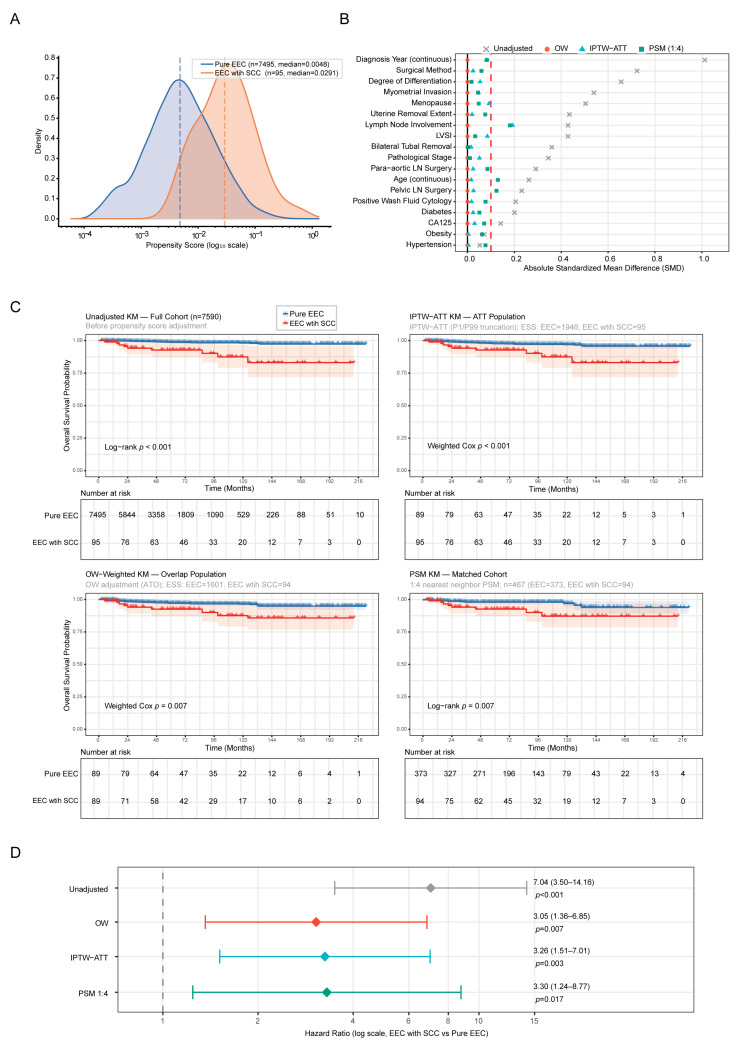
Propensity score analysis and confounding-adjusted overall survival. (**A**) PS distributions for EEC with an SCC component and pure EEC; dashed lines indicate median PS values. (**B**) Love plot showing absolute SMDs before and after OW, IPTW-ATT, and 1:4 PSM; the dashed line indicates the balance threshold of 0.10. (**C**) Kaplan–Meier OS curves for the unadjusted full cohort, OW-weighted overlap population, IPTW-ATT-weighted ATT population, and PSM-matched cohort; shaded areas indicate 95% CIs and tick marks indicate censoring. (**D**) Forest plot of aHRs from doubly robust Cox regression incorporating PS-based weighting or matching and a multivariable outcome model; diamonds and horizontal lines indicate aHRs and 95% CIs, respectively, and the dashed line indicates an aHR of 1.0.

PS coefficients are in Supplementary Table S3 (Nagelkerke R^2^ = 0.182; AIC = 900.81). EEC with SCC component was associated with earlier diagnosis year (OR 0.54), G3 differentiation (OR 2.55), deep myometrial invasion (OR 1.58), laparotomy (OR 1.93), and pelvic lymph node dissection (OR 2.27; all *p* ≤ 0.05). Diagnosis year was included to address earlier-era enrichment. Common support was adequate (0.003–0.650; 4605/7495 pure EEC [61.4%]) (Figure 2A).

Before adjustment, 16/18 covariates had |SMD| ≥ 0.1 (mean |SMD| = 0.383) (Figure 2B; Supplementary Table S4). After OW, all covariates balanced exactly (SMD = 0.000; effective sample size [ESS]: 1601 and 94.1). After IPTW-ATT, 17/18 covariates had SMD < 0.1 (mean SMD = 0.045; ESS: 1946 and 95), with residual LN imbalance (SMD = 0.193). After 1:4 PSM, 94/95 SCC-component patients matched (*n* = 467), with residual imbalance in LN involvement, age, and pelvic lymph node surgery.

Kaplan–Meier analysis showed worse OS for EEC with SCC component (log-rank *p* < 0.001), persisting after PS adjustment (OW *p* = 0.007; IPTW-ATT *p* < 0.001; PSM *p* = 0.007; Figure 2C). DR Cox models adjusted for stage, grade, myometrial invasion, age, and residual LN imbalance where needed gave consistent estimates (Figure 2D): OW aHR 3.05 (95% CI 1.36–6.85; *p* = 0.007), IPTW-ATT aHR 3.26 (95% CI 1.51–7.01; *p* = 0.003), and PSM aHR 3.30 (95% CI 1.24–8.77; *p* = 0.017).

Additional calendar-period sensitivity analyses were performed to evaluate potential treatment-era heterogeneity. In the full cohort, additional adjustment for diagnosis year yielded results consistent with the primary analysis (OW-adjusted HR, 3.11; 95% CI, 1.35–7.15; IPTW-ATT-adjusted HR, 3.26; 95% CI, 1.48–7.18; PSM-adjusted HR, 3.31; 95% CI, 1.24–8.84). Diagnosis-period-stratified Cox models showed similar results. In the recent-period analysis restricted to 2005–2019, the SCC-component group remained associated with worse OS in the minimally adjusted model (HR, 2.70; 95% CI, 1.21–6.03) and after further adjustment for diagnosis year (HR, 2.49; 95% CI, 1.09–5.68). More recent cutoffs were limited by sparse SCC-component deaths and were considered descriptive or not estimable (Supplementary Table S5).

### 3.4. Secondary Analysis of Lymph Node Involvement

For LN involvement, EEC with SCC component showed a nonsignificant crude trend toward higher LN positivity (OR 1.716; 95% CI 0.885–3.330; *p* = 0.110), which disappeared after OW (OR 1.000; 95% CI 0.505–1.979; *p* = 1.000) and remained null after IPTW-ATT (OR 1.035; 95% CI 0.527–2.035; *p* = 0.920; Supplementary Table S6). Because only 10 SCC-component cases had LN positivity, insufficient power remains a plausible explanation for the null-adjusted association, and a moderate LN association cannot be excluded.

### 3.5. Machine-Learning Survival Performance

Five models were trained (*n* = 7590) (Table 3; Supplementary Table S7). RSF had the highest test C-index (0.817 ± 0.065) and 1-year AUC (0.938 ± 0.045). XGBoost had similar discrimination (test C-index 0.797 ± 0.057) and highest 5-year AUC (0.787 ± 0.065). GBSA declined at 5 years, while Lasso-Cox and CoxPH were lower, consistent with the small number of OS events and severe class imbalance. Brier scores were low. XGBoost was selected for SHAP; median-risk KM stratification separated groups (*p* < 0.001; Supplementary Figure S2).

### 3.6. SHAP Interpretation and Downsampling Sensitivity Analysis

At the original 79:1 ratio, histological subtype ranked 9th of 25 features (mean |SHAP| = 0.273) (Supplementary Figure S3A); the beeswarm plot showed positive SHAP values in EEC with SCC component but near-zero clustering in pure EEC, indicating class-imbalance dilution (Supplementary Figure S3B).

**Figure 3:**
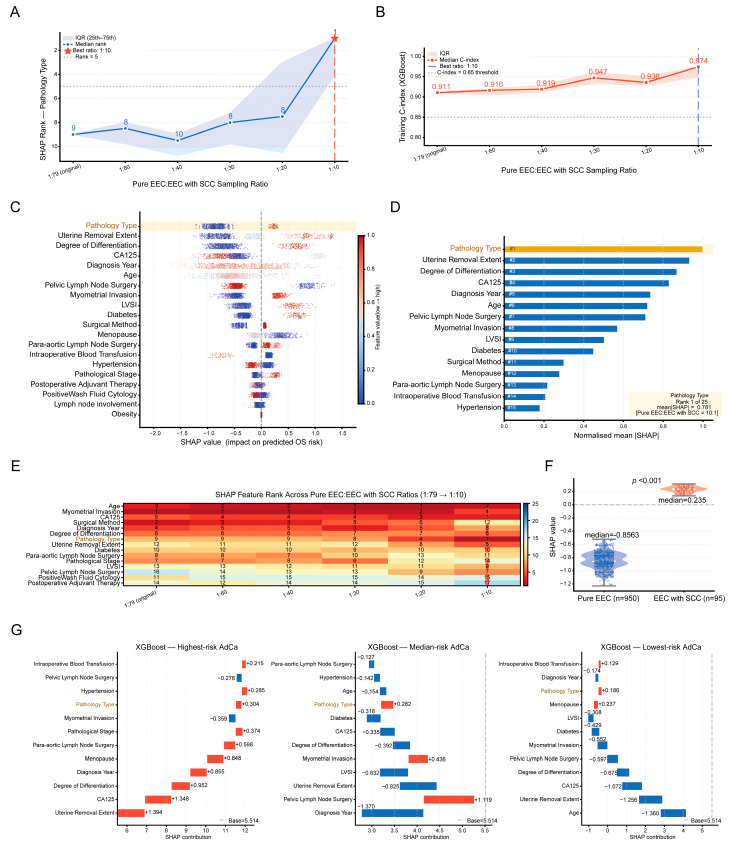
SHAP feature importance and gradient downsampling sensitivity analysis. Positive SHAP values indicate higher model-predicted mortality risk. Downsampling was used only as an imbalance-sensitivity analysis to evaluate whether the rare SCC-component category was diluted in the original 79:1 cohort ratio; it was not used to train a final clinical prediction model. (**A**) Median SHAP rank of histological subtype across six progressive pure EEC:EEC-with-SCC downsampling ratios. (**B**) Median training C-index (XGBoost-Survival) at each downsampling ratio across 10 replicates. (**C**) Global SHAP beeswarm plot at the optimal 10:1 ratio, showing individual-patient SHAP values for the top-ranked features. (**D**) Bar chart of normalized mean absolute SHAP values at the 10:1 ratio, with feature ranks annotated. (**E**) Feature rank heatmap across all six downsampling ratios. (**F**) Violin plot comparing histological-subtype SHAP value distributions between pure EEC and EEC with SCC component at the 10:1 ratio. (**G**) Patient-level waterfall plots for three representative EEC with SCC component patients (highest-, median-, and lowest-predicted-risk). Bars indicate individual feature SHAP contributions; orange bars = positive (risk-increasing), blue bars = negative (risk-decreasing).

Downsampling was used only as an imbalance-sensitivity analysis, not as a final model-training strategy. It used six ratios (79:1 to 10:1; Supplementary Table S8). Median histological-subtype rank was 9, 8.5, 9.5, 8.0, 7.5, and 1.0, with C-index 0.911, 0.916, 0.919, 0.947, 0.936, and 0.974 (Figure 3A,B). At 10:1, histological subtype ranked first in 7/10 replicates (70%; mean |SHAP| = 0.781), surpassing uterine removal extent, degree of differentiation, and CA-125; the rank heatmap showed overall improvement toward the 10:1 ratio (Figure 3C–E).

At 10:1, histological-subtype SHAP distributions separated (Figure 3F; Mann–Whitney *p* < 0.001): median +0.235 in EEC with SCC component versus −0.856 in pure EEC. Waterfall plots (Figure 3G) showed positive contributions in representative patients. Thus, the low 79:1 rank reflected dilution, and EEC with SCC component became a prominent risk-associated feature after cohort composition was addressed.

### 3.7. Exploratory Analysis of EEC with SCC Component

Within EEC with SCC component (*n* = 95; 9 OS events), exploratory univariate Cox regression identified elevated CA-125 (HR 5.63), vaginal discharge (HR 6.64), and pelvic lymph node dissection (HR 0.24) as *p* < 0.05 variables (Supplementary Table S9). The lymph node dissection signal likely reflects selection bias. Nonsignificant trends appeared for deep myometrial invasion and G3 grade; several variables showed complete-separation nonconvergence. Kaplan–Meier curves are shown in Supplementary Figure S4 (log-rank *p* = 0.020, 0.001, and 0.020).

## 4. Discussion

In this multicenter cohort, EEC with SCC component was rare but showed worse crude OS than pure EEC. After PS/DR adjustment, it remained associated with an approximately three-fold higher mortality hazard, suggesting prognostic information beyond available clinicopathological factors used in routine risk assessment. This association should be interpreted cautiously because the SCC-component group included only 95 patients and 9 OS deaths, resulting in wide confidence intervals despite consistent direction across PS strategies. Machine-learning survival modeling with SHAP supported this finding after addressing class imbalance.

For LN involvement, the crude trend toward higher positivity disappeared after OW and IPTW-ATT, suggesting that the OS disadvantage is unlikely to be driven by a measured increase in LN metastasis after adjustment. This matters because nodal risk guides staging and adjuvant decisions. However, the SCC-component subgroup included only 10 LN-positive cases, so insufficient power remains a plausible explanation for the null adjusted association.

Rare aggressive phenotypes are confounding-prone because histology co-segregates with grade, myometrial invasion, stage, treatment intensity, and diagnostic era. PS methods balance measured covariates before outcome modeling [23]. We therefore used complementary OW, IPTW-ATT, and 1:4 PSM approaches under extreme imbalance, followed by DR Cox regression to improve robustness to model misspecification [23,27,30]. The attenuation from a crude HR of about 7 to adjusted aHRs of about 3 indicates substantial confounding, while the persistent residual association supports a robust adverse association after adjustment.

Machine-learning survival modeling served as an exploratory interpretability scaffold rather than a deployable prediction tool. XGBoost was selected for SHAP because it provided strong long-horizon discrimination and exact TreeSHAP attribution [28,29]. Given the rarity of EEC with SCC component (~1:79), gradient downsampling tested class-imbalance dilution, consistent with oncology machine-learning practice for minority-class prognostic signals [31].

Across WHO editions, squamous differentiation has been treated as part of the EEC morphologic spectrum rather than a stand-alone entity. WHO 2003 listed a “variant with squamous differentiation,” WHO 2014 retained it within the endometrioid category, and WHO 2020 continued to regard it as an optional morphologic feature within EEC [6,14,17]. This is consistent with FIGO grading, which excludes squamous differentiation from the solid non-glandular, non-squamous growth fraction used for grading; squamous differentiation is also not a staging criterion [32]. Thus, benign-appearing squamous differentiation, including morules, has generally been handled descriptively [17]. However, the WHO category of EEC with squamous differentiation has historically also encompassed tumors with SCC components, previously labeled adenosquamous carcinoma. For this reason, the analytic exposure in the present study was not any squamous differentiation, but an explicitly recorded SCC component in institutional final pathology reports and retained after standardized database-level reclassification. This study-specific wording distinguishes carcinoma-level squamous components from benign squamous differentiation, squamous metaplasia, morular differentiation, and adenoacanthoma-like terminology within the broader WHO framework; it is not intended to propose a new WHO histotype. Our adjusted results suggest this distinction is clinically relevant: EEC with SCC component showed an adverse OS association, indicating prognostic information not fully captured by grade and stage.

This finding does not imply more extensive nodal surgery. WHO 2020 recognizes primary endometrial SCC as a rare entity and notes stage-dependent behavior for squamous carcinomas, whereas NCCN nodal assessment remains based on established clinicopathological risk features [3,17,33]. Consistently, our PS-weighted analysis found no adjusted increase in LN involvement for EEC with SCC component. Thus, EEC with SCC component should not trigger expanded nodal procedures alone; instead, it should be explicitly reported as a potentially adverse morphologic feature and considered in postoperative risk discussion, especially when molecular profiling is incomplete.

The null-adjusted LN finding should also be interpreted in relation to invasion-pattern information that was not standardized in this registry. MELF (microcystic, elongated, and fragmented) invasion is an invasive-front pattern in endometrioid carcinoma that has been associated with LVSI, nodal spread, and locally aggressive invasion [34,35,36]. Recent data further suggest that squamous differentiation may coexist with MELF and identify cases with higher nodal-metastasis ratios [37]. Because MELF presence, extent, and its spatial relationship to the SCC component were not captured as registry fields, we could not directly test whether MELF-related invasion confounded or mediated the SCC-component association. Future registries should record MELF, LVSI extent, SCC-component proportion, and nodal status in a standardized manner.

Our findings help reconcile inconsistent reports on EEC with malignant-appearing squamous components, historically termed adenosquamous carcinoma [9,12,13]. Prior studies were small, single-center series with evolving thresholds and limited adjustment [10,12,13,21,22]. This multicenter analysis provides a larger adjusted estimate of the SCC-component prognostic association within EEC.

Cross-organ evidence supports plausibility [38]. Adenosquamous carcinoma is more aggressive than corresponding adenocarcinoma in the pancreas [39], lung [40], and cervix [41]. Although these analogies cannot substitute for endometrial-specific evidence or define effect size, they support the concept that malignant squamous lineage programs may carry adverse prognostic implications within gland-forming carcinomas.

The molecular era interpretation of an SCC component requires caution. TCGA/ProMisE groups have distinct outcomes, from favorable POLE-mutated tumors to adverse p53-abnormal disease [15,16,42,43,44]. An SCC component may therefore represent a morphology-linked risk signal within molecularly heterogeneous EEC, enrichment for p53-abnormal or other high-risk biology, or a surrogate for an unmeasured molecular subgroup. Several mechanisms could plausibly link an SCC component with worse outcome. Primary endometrial SCC often expresses p40/p63 and CK5/6 with absent or focal Müllerian markers (ER/PR, PAX8) [33]. An SCC component may also reflect clonal evolution or dedifferentiation, TP53-abnormal biology and/or SWI/SNF disruption such as ARID1A loss, and keratinizing tumor programs related to hypoxia, inflammation, necrosis, or reduced treatment sensitivity [44,45,46,47,48]. These mechanisms remain hypothesis-generating because molecular subtype, SCC-component proportion, and confirmatory IHC data were not systematically available. Because POLE, MMR, p53, and NSMP assignments were incomplete, the present results show an association after available clinicopathological adjustment but do not establish independence from molecular subtype.

Our analyses used FIGO 2009 stage [24], with stage entered as a broad I/II versus III/IV covariate. In the current molecular era, these findings should be interpreted in the context of FIGO 2023. FIGO 2023 incorporates molecular classification and LVSI extent, and POLEmut- or p53abn-modified stage categories require systematic POLE/MMR/p53 data [5,49,50]. Reliable patient-level FIGO 2023 restaging was not possible in this historical multicenter registry because molecular testing was not uniformly available across the 2000–2019 accrual period and LVSI was not consistently recorded in the FIGO 2023 focal/substantial-extensive format. Although most changes for EEC would refine early-stage subdivisions, selected scenarios, such as low-grade EEC with ovarian involvement meeting IA3 criteria, could cross the broad I/II versus III/IV boundary used in our models. Therefore, we did not rerun the models by FIGO 2023 stage; instead, we interpret the present findings as FIGO 2009/available-clinicopathological-adjusted associations that require future validation in cohorts with systematic molecular testing and FIGO 2023-compatible LVSI quantification.

Clinically, when molecular profiling is unavailable or incomplete, EEC with SCC component may represent a high-risk morphologic signal warranting documentation and multidisciplinary discussion. Reporting SCC-component proportion may improve reproducibility and threshold-based modeling. This finding should not by itself justify more extensive nodal surgery or a specific adjuvant regimen, but it supports explicit pathology reporting and future validation in molecularly classified cohorts. Elevated CA-125 and vaginal discharge are hypothesis-generating; the apparent lymph node dissection benefit likely reflects selection bias.

Strengths include the largest multicenter EEC with SCC component cohort reported to date, a WHO 2014-based standardized database-level reclassification from institutional final pathology reports, and consistent findings across three PS strategies with DR Cox modeling. ML survival modeling with SHAP added interpretable support while addressing class-imbalance dilution, complementing conventional clinicopathological risk assessment and motivating molecular validation.

Limitations include few deaths overall (81) and within EEC with an SCC component (9), resulting in wide confidence intervals and exploratory within-subtype modeling. Pathology classification was based on institutional final pathology reports and standardized electronic field-level reclassification after source-document abstraction, local cross-checking, and central field-level review. Centralized slide-level pathology review, study-level immunohistochemical confirmation using original stains, SCC-component proportion/grading/scoring, and formal slide-level interobserver reproducibility assessment would require a separate pathology validation design with access to original or digital slides and were not available in the current registry-based study. In addition, molecular and immunohistochemical data were incompletely available. This historical multicenter cohort spans 2000–2019, a period during which molecular classification and guideline-based molecular testing were progressively incorporated into clinical practice. Therefore, POLE mutation status and complete MMR/p53 data were not systematically available across centers and calendar years, particularly in the EEC with SCC-component subgroup. Although available registry-derived fields included MMR-related markers such as MLH1, MSH2, MSH6, and PMS2, together with POLE mutation testing and p53 IHC when recorded, these data were insufficient to support reliable ProMisE/TCGA surrogate molecular classification or molecular subtype-adjusted survival modeling [15,16,42,43,44]. Molecular information was therefore considered descriptively and in the interpretation of residual uncertainty, rather than incorporated into the primary models. Residual confounding may persist because of unmeasured chemotherapy details, comorbidity severity, molecular subtype, and p53 status. Longer follow-up and earlier diagnosis year suggest enrichment from earlier treatment eras; diagnosis year was included in the propensity-score model, and additional calendar-period sensitivity analyses were directionally consistent, but treatment-era heterogeneity cannot be fully eliminated. Finally, histologic review was anchored to WHO 2014 and staging to FIGO 2009, both of which differ from newer molecular era paradigms.

## 5. Conclusions

EEC with SCC component is rare but associated with worse OS after adjustment for available clinicopathological factors. This risk is not explained by an adjusted increase in LN involvement, supporting possible non-nodal aggressiveness. Together, PS/DR and ML/SHAP findings support this phenotype as a relevant morphologic risk signal that merits explicit pathology reporting, particularly where molecular testing is not routine. Available data could not determine whether the association was independent of molecular subtype. Future multicenter studies or registries could standardize SCC-component thresholds, define immunohistochemical and molecular panels, harmonize endpoints, and enable validation.

## Figures and Tables

**Table 1:** Baseline characteristics of patients with pure EEC and EEC with SCC component.

Characteristic	Total (*N* = 7590)	Pure EEC (*n* = 7495)	EEC with SCC Component (*n* = 95)	*p*-Value	SMD
Demographics and risk factors					
Age	53.9 ± 9.2	53.9 ± 9.2	56.3 ± 8.7	0.024	0.262
Diagnosis Year	2013.7 ± 3.5	2013.7 ± 3.5	2010.2 ± 4.0	<0.001	1.013
Diabetes				0.074	0.201
No	6648 (87.6%)	6571 (87.7%)	77 (81.1%)		
Yes	942 (12.4%)	924 (12.3%)	18 (18.9%)		
Hypertension				0.687	0.052
No	4428 (58.3%)	4375 (58.4%)	53 (55.8%)		
Yes	3162 (41.7%)	3120 (41.6%)	42 (44.2%)		
Obesity				0.596	0.071
No	6699 (88.3%)	6613 (88.2%)	86 (90.5%)		
Yes	891 (11.7%)	882 (11.8%)	9 (9.5%)		
Menopause				0.028	0.504
Postmenopausal	4346 (58.7%)	4285 (58.6%)	61 (70.9%)		
Premenopausal	3052 (41.3%)	3027 (41.4%)	25 (29.1%)		
Unknown	192	183	9		
Clinical presentation and laboratory marker					
CA125				0.404	0.142
Elevated (≥35)	1262 (24.1%)	1244 (24.1%)	18 (29.5%)		
Normal (<35)	3966 (75.9%)	3923 (75.9%)	43 (70.5%)		
Unknown	2362	2328	34		
Abnormal Vaginal Bleeding				0.097	0.184
No	4603 (60.7%)	4537 (60.6%)	66 (69.5%)		
Yes	2981 (39.3%)	2952 (39.4%)	29 (30.5%)		
Unknown	6	6	0		
Postmenopausal Vaginal Bleeding				0.032	0.234
No	3744 (49.4%)	3708 (49.5%)	36 (37.9%)		
Yes	3840 (50.6%)	3781 (50.5%)	59 (62.1%)		
Unknown	6	6	0		
Vaginal Discharge				0.012	0.279
No	6844 (90.2%)	6766 (90.3%)	78 (82.1%)		
Yes	740 (9.8%)	723 (9.7%)	17 (17.9%)		
Unknown	6	6	0		
Abdominal Pain				0.048	0.224
No	6872 (90.6%)	6792 (90.7%)	80 (84.2%)		
Yes	712 (9.4%)	697 (9.3%)	15 (15.8%)		
Unknown	6	6	0		
Abdominal Distension				0.195	0.152
No	7370 (97.2%)	7280 (97.2%)	90 (94.7%)		
Yes	214 (2.8%)	209 (2.8%)	5 (5.3%)		
Unknown	6	6	0		
Tumor/pathologic parameters					
Degree of Differentiation				<0.001	0.656
G1/G2	6462 (85.1%)	6403 (85.4%)	59 (62.1%)		
G3	1128 (14.9%)	1092 (14.6%)	36 (37.9%)		
Myometrial Invasion				<0.001	0.539
≤1/2	5671 (74.7%)	5622 (75.0%)	49 (51.6%)		
>1/2	1919 (25.3%)	1873 (25.0%)	46 (48.4%)		
LVSI				1	0.429
No	6106 (93.4%)	6042 (93.4%)	64 (94.1%)		
Yes	434 (6.6%)	430 (6.6%)	4 (5.9%)		
Unknown	1050	1023	27		
Lymph node involvement				0.159	0.429
No	7049 (93.3%)	6967 (93.4%)	82 (89.1%)		
Yes	505 (6.7%)	495 (6.6%)	10 (10.9%)		
Unknown	36	33	3		
Pathological Stage				0.001	0.345
Stage I/II	6732 (88.7%)	6658 (88.8%)	74 (77.9%)		
Stage III/IV	858 (11.3%)	837 (11.2%)	21 (22.1%)		
Surgical management					
Surgical Method				<0.001	0.724
Laparoscopy	4139 (54.5%)	4121 (55.0%)	18 (18.9%)		
Laparotomy	3451 (45.5%)	3374 (45.0%)	77 (81.1%)		
Uterine Removal Extent				0.036	0.435
Extra fascial	4426 (67.1%)	4374 (67.3%)	52 (55.9%)		
Modified Radical	946 (14.3%)	925 (14.2%)	21 (22.6%)		
Radical	1225 (18.6%)	1205 (18.5%)	20 (21.5%)		
Unknown	993	991	2		
Bilateral Tubal Removal				<0.001	0.36
No	1783 (23.5%)	1775 (23.7%)	8 (8.4%)		
Yes	5807 (76.5%)	5720 (76.3%)	87 (91.6%)		
Ovarian Preservation				0.614	0.075
Preserved	452 (6.0%)	448 (6.0%)	4 (4.2%)		
Removed	7138 (94.0%)	7047 (94.0%)	91 (95.8%)		
Pelvic Lymph Node Surgery				0.081	0.232
Biopsy	199 (2.6%)	197 (2.6%)	2 (2.1%)		
Dissection	5458 (71.9%)	5380 (71.8%)	78 (82.1%)		
No	1933 (25.5%)	1918 (25.6%)	15 (15.8%)		
Para-aortic Lymph Node Surgery				0.019	0.292
Biopsy	947 (12.5%)	927 (12.4%)	20 (21.1%)		
Dissection	2420 (31.9%)	2398 (32.0%)	22 (23.2%)		
No	4223 (55.6%)	4170 (55.6%)	53 (55.8%)		
Perioperative/postoperative events					
Surrounding Organ Injury				1	0.066
No	7558 (99.6%)	7463 (99.6%)	95 (100.0%)		
Yes	32 (0.4%)	32 (0.4%)	0 (0.0%)		
Residual Lesion				0.213	0.163
No	7571 (99.7%)	7477 (99.8%)	94 (98.9%)		
Yes	19 (0.3%)	18 (0.2%)	1 (1.1%)		
Intraoperative Blood Transfusion				0.026	0.264
No	7218 (95.1%)	7133 (95.2%)	85 (89.5%)		
Yes	372 (4.9%)	362 (4.8%)	10 (10.5%)		
Postoperative Complications				0.022	0.281
No	7462 (98.3%)	7372 (98.4%)	90 (94.7%)		
Yes	128 (1.7%)	123 (1.6%)	5 (5.3%)		
Positive Wash Fluid Cytology				0.096	0.205
No	4386 (95.3%)	4339 (95.3%)	47 (90.4%)		
Yes	217 (4.7%)	212 (4.7%)	5 (9.6%)		
Unknown	2987	2944	43		
Adjuvant therapy					
Postoperative Adjuvant Therapy				<0.001	0.48
No	4620 (60.9%)	4582 (61.1%)	38 (40.0%)		
Chemoradiotherapy	750 (9.9%)	735 (9.8%)	15 (15.8%)		
Chemotherapy alone	1664 (21.9%)	1637 (21.8%)	27 (28.4%)		
Radiotherapy alone	556 (7.3%)	541 (7.2%)	15 (15.8%)		

Note: EEC, endometrioid endometrial carcinoma; SCC, squamous cell carcinoma; SMD, standardized mean difference; LVSI, lymphovascular space invasion. Unknown values indicate unavailable or indeterminate entries in the retrospective multicenter registry and are shown to transparently report missingness. Percentages were calculated using the total number in each group as the denominator unless otherwise specified.

**Table 2:** Univariate Cox proportional hazards regression for overall survival.

Variable	Category	HR	95% CI	*p*-Value
Histological subtype	Pure EEC	1.00 (reference)		
	EEC with SCC component	7.04	3.50–14.16	<0.001
Age	Per unit increase	1.05	1.03–1.08	<0.001
Diagnosis Year	Per unit increase	0.94	0.89–1.00	0.047
Diabetes	No	1.00 (reference)		
	Yes	2.23	1.34–3.74	0.002
Hypertension	No	1.00 (reference)		
	Yes	1.35	0.87–2.09	0.178
Obesity	No	1.00 (reference)		
	Yes	1.83	1.03–3.25	0.041
Menopause	Premenopausal	1.00 (reference)		
	Postmenopausal	2.47	1.46–4.16	<0.001
	Unknown	0.00	0.00–inf	0.994
CA125	Normal (<35)	1.00 (reference)		
	Elevated (≥35)	3.44	2.12–5.60	<0.001
	Unknown	0.75	0.41–1.38	0.354
Abnormal Vaginal Bleeding	No	1.00 (reference)		
	Yes	0.47	0.28–0.78	0.003
	Unknown	0.00	0.00–inf	0.996
Postmenopausal Vaginal Bleeding	No	1.00 (reference)		
	Yes	1.56	1.00–2.44	0.053
	Unknown	0.00	0.00–inf	0.996
Vaginal Discharge	No	1.00 (reference)		
	Yes	2.38	1.39–4.06	0.002
	Unknown	0.00	0.00–inf	0.996
Abdominal Pain	No	1.00 (reference)		
	Yes	2.00	1.12–3.56	0.018
	Unknown	0.00	0.00–inf	0.996
Abdominal Distension	No	1.00 (reference)		
	Yes	1.80	0.66–4.91	0.253
	Unknown	0.00	0.00–inf	0.996
Surgical Method	Laparoscopy	1.00 (reference)		
	Laparotomy	4.33	2.32–8.05	<0.001
Uterine Removal Extent	Extra fascial	1.00 (reference)		
	Modified Radical	0.74	0.39–1.44	0.379
	Radical	0.69	0.37–1.29	0.243
	Unknown	0.52	0.21–1.32	0.169
Bilateral Tubal Removal	No	1.00 (reference)		
	Yes	2.75	1.19–6.32	0.017
Ovarian Preservation	Preserved	1.00 (reference)		
	Removed	1.18	0.48–2.94	0.72
Pelvic Lymph Node Surgery	No	1.00 (reference)		
	Biopsy	0.00	0.00–inf	0.993
	Dissection	1.58	0.91–2.74	0.102
Para-aortic Lymph Node Surgery	No	1.00 (reference)		
	Biopsy	2.10	1.22–3.64	0.008
	Dissection	0.95	0.55–1.66	0.865
Surrounding Organ Injury	No	1.00 (reference)		
	Yes	7.09	1.74–28.85	0.006
Residual Lesion	No	1.00 (reference)		
	Yes	6.70	0.93–48.18	0.059
Intraoperative Blood Transfusion	No	1.00 (reference)		
	Yes	2.61	1.45–4.71	0.001
Postoperative Complications	No	1.00 (reference)		
	Yes	1.38	0.34–5.62	0.653
Positive Wash Fluid Cytology	No	1.00 (reference)		
	Yes	3.57	1.61–7.90	0.002
	Unknown	0.84	0.52–1.35	0.474
Degree of Differentiation	G1/G2	1.00 (reference)		
	G3	4.14	2.66–6.43	<0.001
Myometrial Invasion	≤1/2	1.00 (reference)		
	>1/2	4.63	2.94–7.31	<0.001
LVSI	No	1.00 (reference)		
	Yes	3.74	1.95–7.16	<0.001
	Unknown	1.45	0.82–2.56	0.205
Lymph node involvement	No	1.00 (reference)		
	Yes	5.54	3.36–9.13	<0.001
	Unknown	6.51	1.59–26.66	0.009
Pathological Stage	Stage I/II	1.00 (reference)		
	Stage III/IV	7.14	4.61–11.05	<0.001
Postoperative Adjuvant Therapy	No	1.00 (reference)		
	Radiotherapy alone	1.48	0.62–3.54	0.377
	Chemotherapy alone	1.87	1.09–3.22	0.024
	Chemoradiotherapy	4.12	2.38–7.15	<0.001

Note: EEC, endometrioid endometrial carcinoma; SCC, squamous cell carcinoma; HR, hazard ratio; CI, confidence interval.

**Table 3:** Cross-validated performance metrics of five survival machine learning models.

Metric	Train C-Index	Test C-Index	AUC 1 yr	AUC 3 yr	AUC 5 yr	BS 1 yr	BS 3 yr	BS 5 yr
CoxPH	0.738 ± 0.027	0.721 ± 0.065	0.742 ± 0.292	0.776 ± 0.076	0.599 ± 0.085	0.002 ± 0.002	0.010 ± 0.002	0.018 ± 0.004
Lasso-Cox	0.781 ± 0.024	0.776 ± 0.098	0.770 ± 0.184	0.614 ± 0.078	0.580 ± 0.074	0.002 ± 0.001	0.007 ± 0.001	0.014 ± 0.003
RSF	0.970 ± 0.003	0.817 ± 0.065	0.938 ± 0.045	0.816 ± 0.048	0.736 ± 0.084	0.002 ± 0.001	0.008 ± 0.001	0.015 ± 0.003
XGBoost	0.921 ± 0.011	0.797 ± 0.057	0.726 ± 0.261	0.791 ± 0.034	0.787 ± 0.065	0.002 ± 0.001	0.007 ± 0.001	0.014 ± 0.003
GBSA	0.778 ± 0.022	0.752 ± 0.048	0.865 ± 0.075	0.780 ± 0.043	0.614 ± 0.069	0.002 ± 0.001	0.007 ± 0.001	0.015 ± 0.003

Note: AUC, time-dependent area under the ROC curve; BS, Brier score; CoxPH, Cox proportional hazards; Lasso-Cox, Lasso-penalized Cox; RSF, random survival forest; GBSA, gradient boosting survival analysis.

## Data Availability

The data presented in this study are available from the corresponding author on reasonable request due to privacy and ethical restrictions.

## Supplementary Materials

The following supporting information can be downloaded at: https://www.mdpi.com/article/10.3390/cancers18142275/s1, Figure S1. Covariate selection process for the propensity score model. Figure S2. XGBoost-Survival Kaplan–Meier risk stratification. Figure S3. Global XGBoost-Survival SHAP analysis at the original cohort ratio. (A) Bar chart of mean absolute SHAP values (mean |SHAP|) for all 25 features ranked by importance. (B) Beeswarm plot showing individual-patient SHAP values for the top 15 features. Figure S4. Kaplan–Meier overall survival curves for significant prognostic factors within the EEC with SCC component subgroup. (A) CA-125 level (<35 vs. ≥35 U/mL). (B) Vaginal discharge at presentation (absent vs. present). (C) Pelvic lymph node dissection (not performed vs. performed); Table S1. Standardized mapping of historical pathology-report diagnostic terms to analytic histological categories. Table S2. Variance inflation factors for propensity score model covariates. Table S3. Propensity score model coefficients and model fit statistics. Table S4. Absolute standardized mean differences before and after propensity score adjustment. Table S5. Calendar-period sensitivity analyses for overall survival. Table S6. Propensity score–weighted logistic regression for lymph node involvement. Table S7. Per-fold cross-validation performance metrics for all five survival machine learning models. Table S8. Gradient downsampling sensitivity analysis: histological-subtype SHAP rank and C-index by ratio and replicate. Table S9. Univariate Cox proportional hazards regression within the EEC with SCC component subgroup. Supplementary methods.

## Abbreviations

The following abbreviations are used in this manuscript:

AUC	time-dependent area under the ROC curve
ATT	average treatment effect on the treated
ATO	average treatment effect in the overlap population
aHR	adjusted hazard ratio
CI	confidence interval
C-index	Harrell’s concordance index
CoxPH	Cox proportional hazards model
DR	doubly robust (estimation/modeling)
EEC	endometrioid endometrial carcinoma
EPV	events per variable
ESS	effective sample size
FIGO	International Federation of Gynecology and Obstetrics
GBSA	gradient boosting survival analysis
HC0	Huber–White robust (sandwich) variance estimator
HR	hazard ratio
ICD-O	International Classification of Diseases for Oncology
IQR	interquartile range
IPTW	inverse probability of treatment weighting
IPTW-ATT	inverse probability of treatment weighting targeting the ATT
KM	Kaplan–Meier
LN	lymph node
LVSI	lymph-vascular space invasion
MMR	mismatch repair
MSI	microsatellite instability
NSMP	no specific molecular profile
OR	odds ratio
OS	overall survival
OW	overlap weighting
POLE	polymerase epsilon
ProMisE	Proactive Molecular Risk Classifier for Endometrial Cancer
PS	propensity score
PSM	propensity score matching
ROC	receiver operating characteristic
RSF	random survival forest
SCC	squamous cell carcinoma
SD	standard deviation
SHAP	SHapley Additive exPlanations
SMD	standardized mean difference
SWI/SNF	SWItch/Sucrose Non-Fermentable chromatin-remodeling complex
TCGA	The Cancer Genome Atlas
TreeSHAP	tree-based SHAP algorithm
VIF	variance inflation factor
WHO	World Health Organization
XGBoost	eXtreme Gradient Boosting
MELF	microcystic, elongated, and fragmented
