# Supplementary material for: Clinical and Prognostic Significance of a Squamous Cell Carcinoma Component in Endometrioid Endometrial Carcinoma: A Multicenter Retrospective Cohort Study
Source: Cancers (Basel). 2026 Jul 15;18(14):2275. doi: 10.3390/cancers18142275 (PMC13406665; doi:10.3390/cancers18142275)
Supplement: Supplementary file 1 [file cancers-18-02275-s001.zip › Supplementary Methods.pdf]

Supplementary Methods

1. Propensity-score model specification

A propensity score for EEC with SCC component versus pure EEC was estimated using a multivariable logistic model. The prespecified covariates were age, menopause, diagnosis year, diabetes, obesity, hypertension, pathological stage, tumor differentiation, myometrial invasion depth, lymphovascular space invasion, lymph node involvement, positive peritoneal washing cytology, CA125, surgical approach, uterine removal extent, bilateral tubal removal, pelvic lymph node surgery, and para-aortic lymph node surgery. Diagnosis year was included to address temporal imbalance across the 2000–2019 accrual period. Symptom variables were excluded because of high subjectivity or recall-bias risk. Post-treatment or perioperative variables, including postoperative adjuvant therapy, intraoperative blood transfusion, postoperative complications, surrounding organ injury, and residual lesion, were excluded to avoid overadjustment or adjustment for potential mediators. Ovarian preservation was excluded because it lacked prognostic significance in univariate screening and was not retained based on the prespecified clinical and statistical criteria. Variance inflation factors were examined to assess collinearity.

2. Propensity-score adjustment and balance assessment

Three propensity-score strategies were used. Overlap weighting (OW) estimated the effect in the overlap population and down-weighted patients with extreme propensity scores. Inverse probability of treatment weighting targeting the average treatment effect on the treated (IPTW-ATT) weighted pure EEC patients to the covariate distribution of the EEC-with-SCC-component group. For IPTW-ATT, pure-EEC weights were winsorized at the 1st and 99th percentiles to reduce the influence of extreme weights. One-to-four nearest-neighbor propensity-score matching (PSM) matched each EEC-with-SCC-component patient to up to four pure EEC patients. For PSM, matching was performed without replacement on the logit propensity-score scale using a caliper of 0.1 SD. Covariate balance was evaluated using absolute standardized mean differences, with values below 0.1 considered acceptable. Weighted or matched Kaplan-Meier curves were used for descriptive survival visualization.

3. Doubly robust survival and lymph-node models

For overall survival, each propensity-score strategy was combined with a multivariable Cox outcome model including pathological stage, tumor grade, myometrial invasion depth, and age. This doubly robust approach was used to reduce sensitivity to misspecification of either the propensity-score or outcome model. For the matched analysis, robust variance estimation clustered by matched set was used. For lymph node involvement, weighted logistic regression with robust sandwich standard errors was used after excluding patients with unknown lymph node status. Results were reported as hazard ratios or odds ratios with 95% confidence intervals.

4. Calendar-period sensitivity analyses

To evaluate treatment-era heterogeneity, additional sensitivity analyses incorporated diagnosis year or calendar period. First, the full-cohort propensity-score/doubly robust Cox models were repeated with diagnosis year additionally included in the outcome model. Second, full-cohort Cox models stratified by diagnosis-period categories were examined. Third, restricted-cohort analyses were performed for patients diagnosed from 2005, 2008, 2010, 2012, or 2014 onward. For the 2005-2019 restricted cohort, Kaplan-Meier analysis, unadjusted Cox regression, minimally adjusted Cox regression, and minimally adjusted Cox regression additionally including diagnosis year were performed. More recent cutoffs were summarized primarily for feasibility because the number of deaths in the EEC-with-SCC-component subgroup became too sparse for stable adjusted modeling.

5. Machine-learning survival models

Five survival models were trained as exploratory model-based sensitivity and interpretability analyses: Cox proportional hazards, Lasso-penalized Cox, random survival forest, gradient boosting survival analysis, and XGBoost-Survival. Twenty-five routinely available clinical and pathological variables were used after excluding symptom variables. Missing values were retained by encoding an Unknown category where applicable. Because the EEC-with-SCC-component group was rare, inverse-frequency class weighting was applied. Models were evaluated using stratified 5-fold cross-validation. Performance metrics included Harrell's C-index, time-dependent AUC, and Brier score. These models were not intended as externally validated clinical prediction tools.

6. SHAP interpretation and downsampling

XGBoost-Survival was selected for SHAP interpretation because it provided strong long-horizon discrimination and allowed exact TreeSHAP attribution. SHAP values were interpreted as model-based feature contributions: positive values indicate increased predicted risk and negative values indicate decreased predicted risk. To assess whether the extreme pure EEC:EEC-with-SCC-component ratio diluted the apparent contribution of histological subtype, gradient downsampling was performed at the original approximate 79:1 ratio and at 60:1, 40:1, 30:1, 20:1, and 10:1 ratios. Each downsampled ratio was repeated 10 times. Feature rank, mean absolute SHAP value, and C-index were summarized across replicates.

7. Software

Statistical analyses were performed using Python and R packages listed in the main manuscript. All tests were two-sided, and $P<0.05$ was considered statistically significant unless otherwise specified.
