# Supplementary figures and images for: Clinical and Prognostic Significance of a Squamous Cell Carcinoma Component in Endometrioid Endometrial Carcinoma: A Multicenter Retrospective Cohort Study
Source: Cancers (Basel). 2026 Jul 15;18(14):2275. doi: 10.3390/cancers18142275 (PMC13406665; doi:10.3390/cancers18142275)

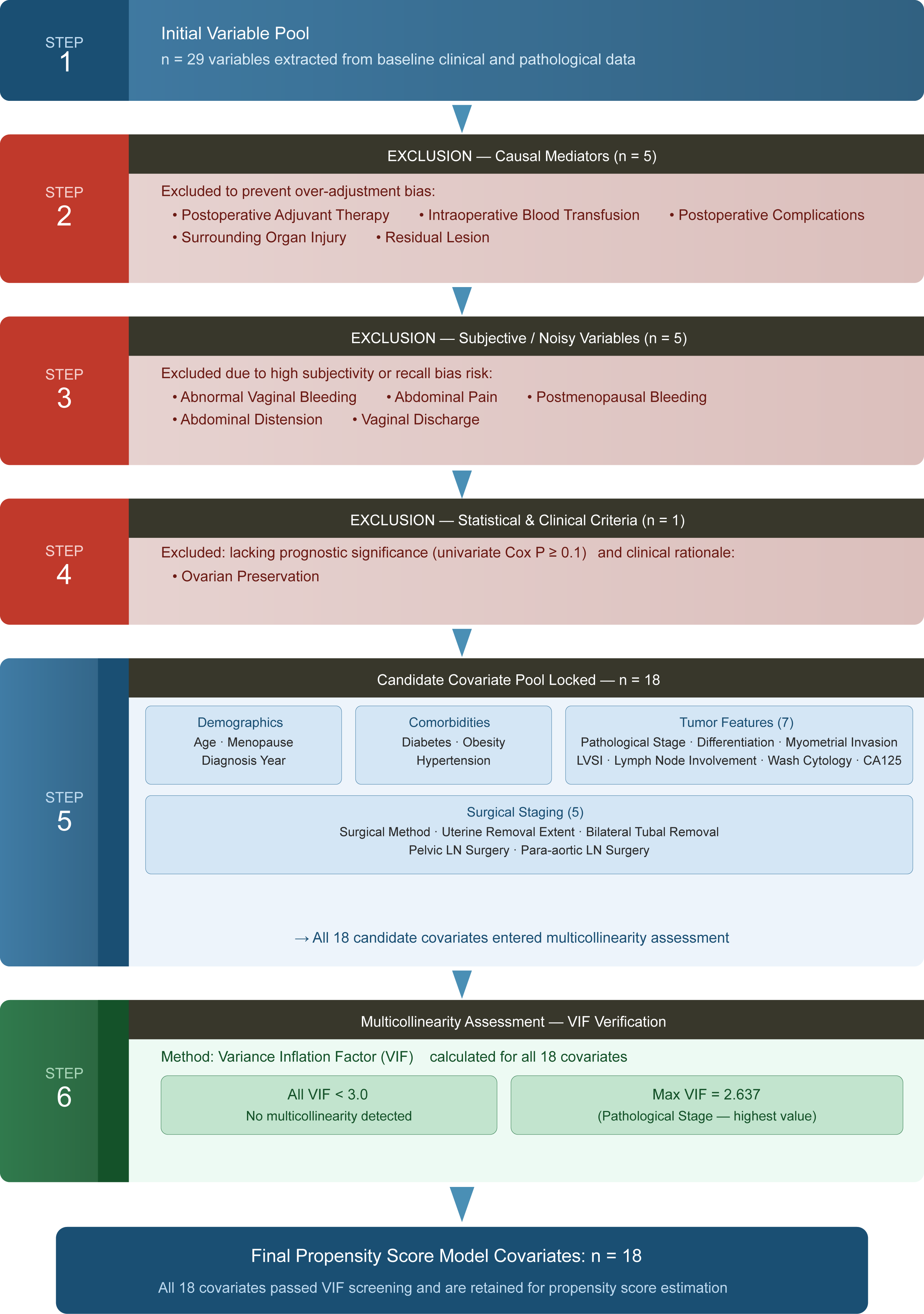

Supplement: Supplementary file 1 [file cancers-18-02275-s001.zip › Supplementary Figure S1.tif]

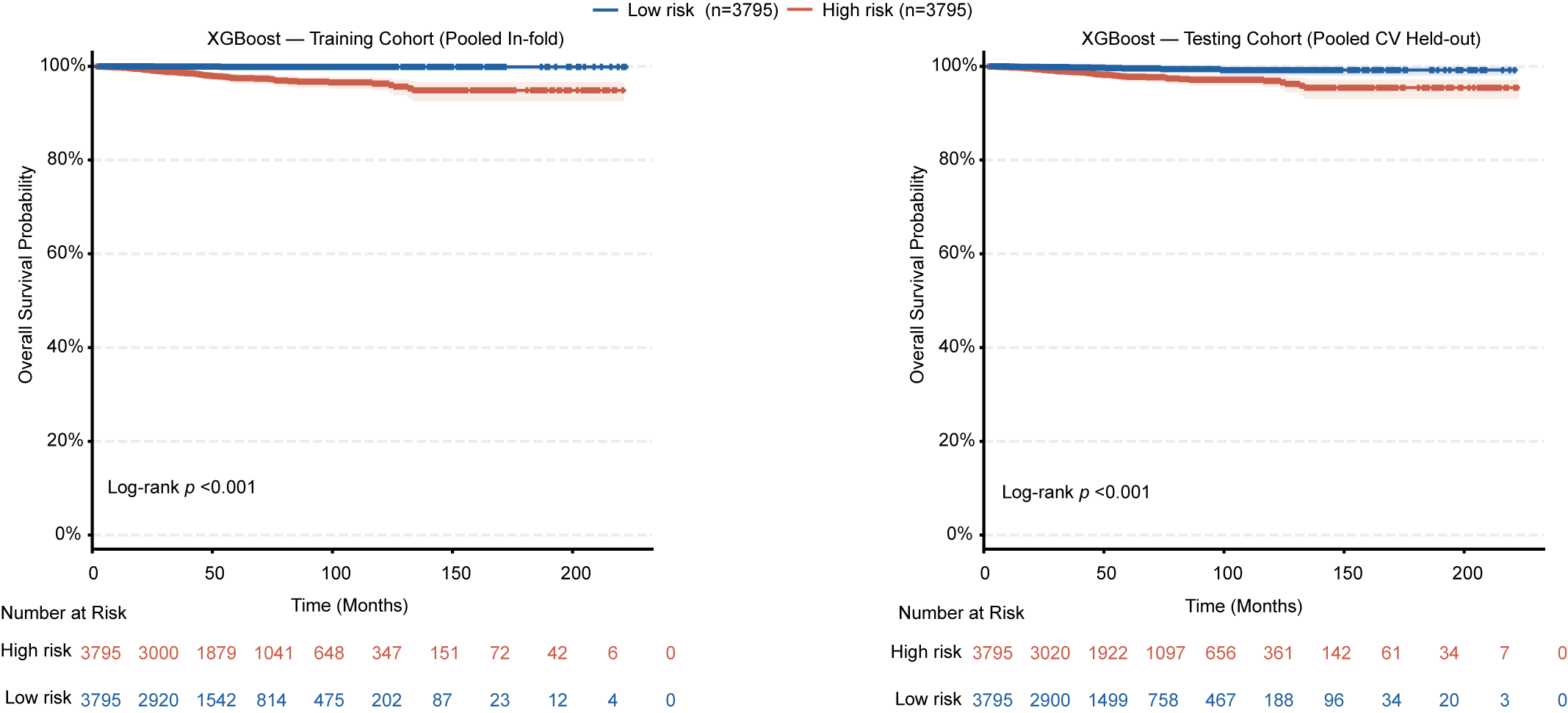

Supplement: Supplementary file 1 [file cancers-18-02275-s001.zip › Supplementary Figure S2.tif]

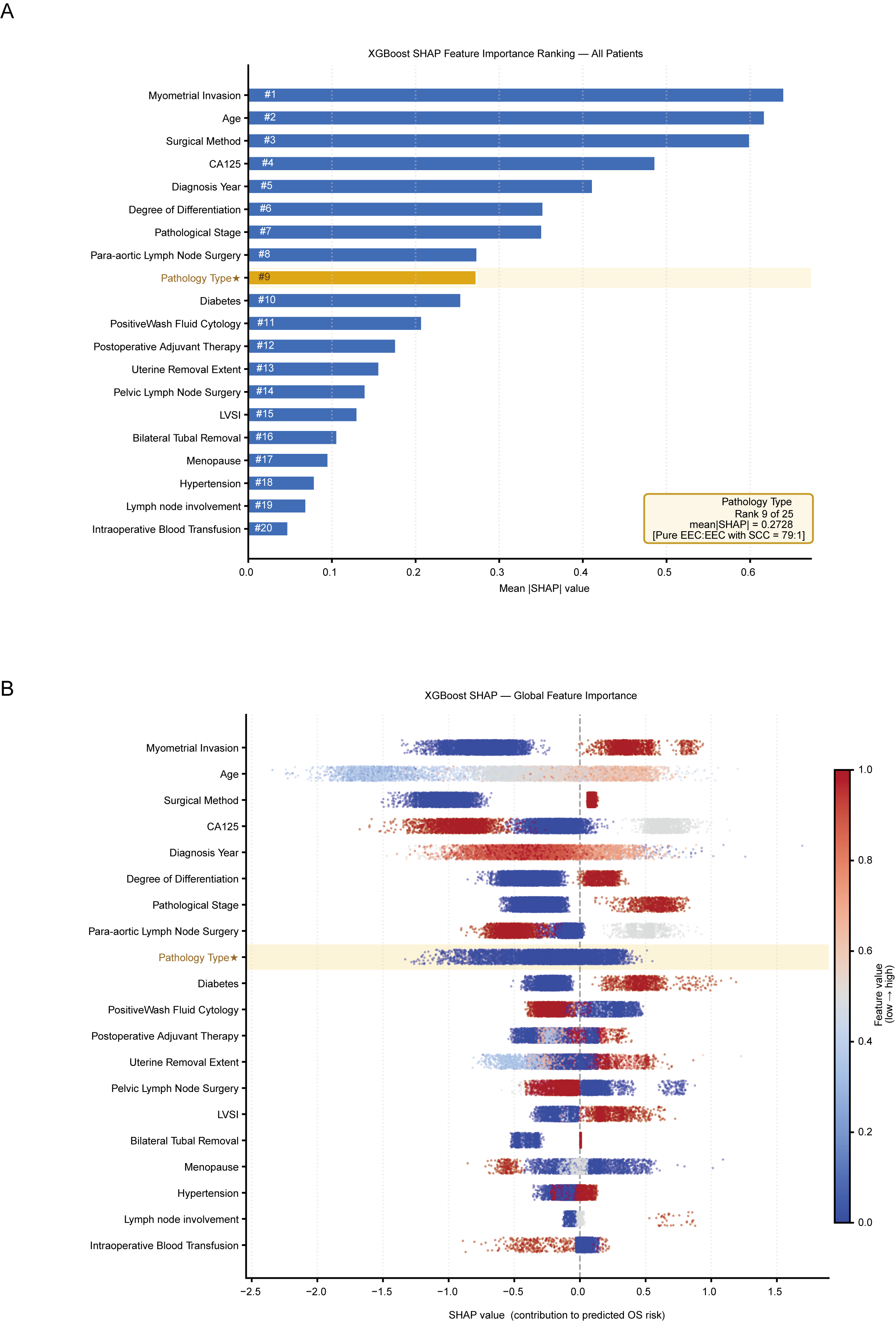

Supplement: Supplementary file 1 [file cancers-18-02275-s001.zip › Supplementary Figure S3.tif]
